# PbS Quantum Dots Saturable Absorber for Dual-Wavelength Solitons Generation

**DOI:** 10.3390/nano11102561

**Published:** 2021-09-29

**Authors:** Ling Yun, Wei Zhao

**Affiliations:** 1 Advanced Photonic Technology Lab, Nanjing University of Posts and Telecommunications, Nanjing 210046, China; yunling@njupt.edu.cn; 2 State Key Laboratory of Transient Optics and Photonics, Xi’an Institute of Optics and Precision Mechanics, Chinese Academy of Sciences, Xi’an 710119, China

**Keywords:** fiber laser, mode locking, PbS quantum dots

## Abstract

PbS quantum dots (QDs), a representative zero-dimensional material, have attracted great interest due to their unique optical, electronic, and chemical characteristics. Compared to one- and two-dimensional materials, PbS QDs possess strong absorption and an adjustable bandgap, which are particularly fascinating in near-infrared applications. Here, fiber-based PbS QDs as a saturable absorber (SA) are studied for dual-wavelength ultrafast pulses generation for the first time to our knowledge. By introducing PbS QDs SA into an erbium-doped fiber laser, the laser can simultaneously generate dual-wavelength conventional solitons with central wavelengths of 1532 and 1559 nm and 3 dB bandwidths of 2.8 and 2.5 nm, respectively. The results show that PbS QDs as broadband SAs have potential application prospects for the generation of ultrafast lasers.

## 1. Introduction

Low-dimensional materials have attracted extensive interest in applied physics due to their excellent optical, electronic, and chemical characteristics [1,2,3]. Two-dimensional (2*D*) graphene [4,5], black phosphorus [6], MXenes [7], antimonene [8], transition metal dichalcogenides [9], topological insulators [10], and one-dimensional (1*D*) carbon nano-tubes [11,12] have been employed as saturable absorbers (SAs) to obtain ultrafast pulses in passively mode-locked fiber lasers. Among these SAs, the main problem is that it is difficult to achieve short carrier lifetime, high thermal damage, large modulation depth, and wide bandwidth in an individual material at the same time. Therefore, one of the ways to solve the problem is to find a new saturable absorption material that can effectively adjust the above parameters.

Semiconductor quantum dots (QDs) are particularly charming materials showing strong quantum confinement effects, as the size of QDs is close to the Bohr radius of the exciton [13]. Strong confinement not only produces interesting new effects but also strengthens the nonlinear optical characteristics [14]. Among numerous semiconductor QDs, PbS QDs possess smaller carrier effective masses and larger optical dielectric constant, leading to a large exciton Bohr radius (~18 nm), which makes relatively large QDs have strong quantum confinement effects [14]. Therefore, combined with the small energy bandgap (~0.41 eV) of PbS, the wavelength range of the excitonic absorption peak is from the visible to 3 μm via changing the size of PbS QDs [15]. This means that PbS QDs can promote the saturable absorption in a large spectral range by changing the size of QDs [16]. As a result, PbS QDs as SAs have been used for Q-switching or mode locking in various near-infrared lasers [13,16,17]. The most obvious advantages of PbS QDs-based SA are adjustable absorption peak, large third-order nonlinear susceptibility, fast response time, large modulation depth, and high damage threshold [18,19].

On the other hand, multi-wavelength passively mode-locked fiber lasers have been investigated extensively in the advancement of fascinating applications as optical fiber sensing, biomedical research, and wavelength division multiplexing (WDM) optical communication [20,21]. Several types of the saturable absorption materials that can realize multi-wavelength passive mode locking have been studied in depth [22,23,24,25,26]. Based on SESAM, Wu et al. realized the dual-wavelength (1553 and 1562 nm) dissipative solitons in Er-doped fiber laser operating at normal dispersion regime [22]. By virtue of carbon nanotube SA, dual-wavelength vector solitons centered at 1533 and 1557 nm were achieved by Zhao et al. [23]. Tunable dual- and triple-wavelength dissipative solitons were obtained from a Yb-doped fiber laser using graphene-oxide mode locker [24]. In a previous paper, we also reported the generation of a dual-wavelength polarization-locked vector solitons fiber laser using black phosphorus SA [25]. However, as far as we know, there is no report of multi-wavelength solitons operating in fiber-based PbS QDs mode-locked fiber lasers.

In this context, PbS QDs are fabricated via a modified hot-injection method. A dual-wavelength passively mode-locked Er-doped fiber laser is realized by using fiber-based PbS QDs as intracavity mode-locked devices. The stable dual-wavelength conventional solitons with central wavelengths of 1532 and 1559 nm, and 3 dB spectral bandwidths of 2.8 and 2.5 nm are obtained. By finely tuning the pump intensity and polarization state, the dual-wavelength mode locking can be switched into single-wavelength operation state.

## 2. Materials and Methods

**Figure 1:**
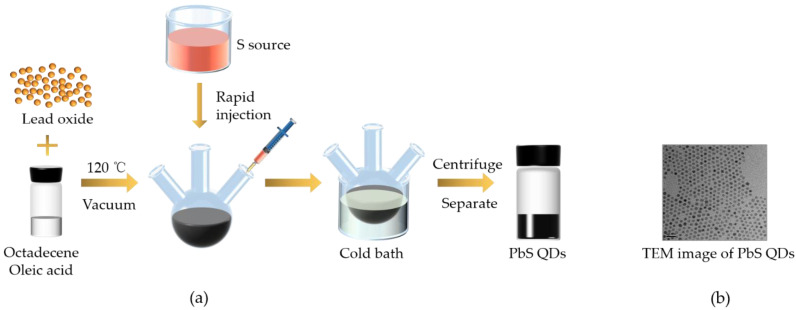
(**a**) The principle diagram of PbS quantum dots (PbS QDs) preparation. (**b**) The transmission electron microscope (TEM) image of PbS QDs.

The PbS QDs coated with oleic acid were prepared by a modified hot-injection meth-od by precisely controlling the mass of the precursor, reaction temperature, environment, and reaction time [18,27]. The preparation details are given in Figure 1a. Firstly, the Pb precursor was formed by putting lead oxide (Sinopharm Chemical Reagent Co., Ltd., Shanghai, China) (450 mg), octadecene (Sinopharm Chemical Reagent Co., Ltd., Shanghai, China) (30 mL), and oleic acid (Sinopharm Chemical Reagent Co., Ltd., Shanghai, China) (10 mL) into a three-necked flask and heating it at 120 °C for 1 h in vacuum. Secondly, the S precursor was prepared by mixing sulfur powder (Sinopharm Chemical Reagent Co., Ltd., Shanghai, China) (32 mg) with oleic amine (Sinopharm Chemical Reagent Co., Ltd., Shanghai, China) (10 mL) and heated to 120 °C until it was completely dissolved. Thirdly, the prepared S precursor was rapidly injected into a three-necked flask containing a Pb source under the protection of argon gas; then, it was cooled to room temperature with an ice water bath quickly. Finally, the sample was separated with ethanol and centrifuged at 12,000 r/min for 3 min. The obtained PbS QDs were dried in vacuum, dissolved in cyclohexane solution (Sinopharm Chemical Reagent Co., Ltd., Shanghai, China), and stored at a concentration of 10 mg/ mL. The transmission electron microscope (TEM) (Hitachi, Tokyo, Japan) image of PbS QDs is given in Figure 1b, which shows that PbS QDs are spherical and have good dispersivity, and the average particle size is ~5.7 nm.

**Figure 2:**
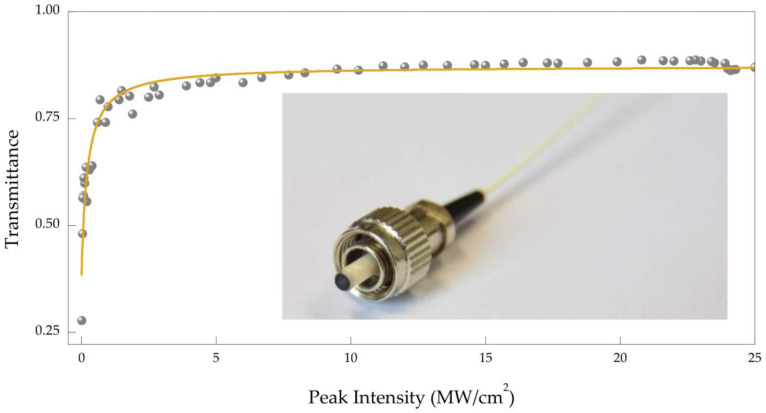
Nonlinear saturable absorption curve of PbS QDs saturable absorber (SA). Inset: PbS QDs mode locker.

The fiber-based PbS QDs mode-locked device was prepared by dropping PbS QDs solution on the end face of an optical fiber connector and evaporated slowly at room temperature and pressure. Compared with the SA prepared by other methods such as mechanical exfoliation or CVD growth, the PbS QDs SA prepared by this scheme avoids the complicated and time-consuming growth and post transfer processes, and it not only overcomes the mechanical damage but also improves the damage threshold of the laser. Based on the dual-path detection system [9,18], the nonlinear optical absorption characteristics of the PbS QDs have been studied. As illustrated in Figure 2, PbS QDs show strong saturable absorption behavior at 1550 nm. The experimental results show that the unsaturable loss, saturation intensity, and modulation depth of the PbS QDs SA are ~21%, ~0.22 MW/cm^2^, and ~44% respectively. To the best of our knowledge, our SA exhibits high modulation depth compared with that reported to date. The corresponding digital photograph of the PbS QDs mode-locker is shown in the inset of Figure 2.

## 3. Results and Discussion

**Figure 3:**
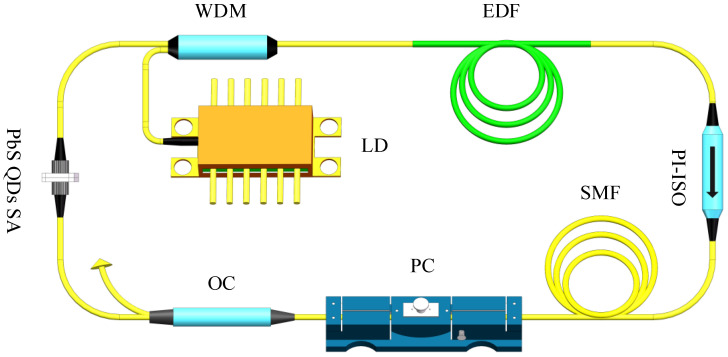
Laser setup. Laser diode (LD), wavelength division multiplexing (WDM), erbium-doped fiber (EDF), polarization-insensitive isolator (PI-ISO), single-mode fiber (SMF), polarization controller (PC), output coupler (OC), and Lead sulfide quantum dots saturable absorber (PbS QDs SA).

The PbS QDs mode-locked Er-doped fiber laser operating in net anomalous dispersion regime is depicted in Figure 3. The ring cavity is composed of a 5.3 m erbium-doped fiber (EDF, *D* = −16 ps/nm/km) and 23.2 m single-mode fiber (SMF, *D* = 17 ps/nm/km). The net cavity dispersion is about −0.39 ps^2^. The EDF served as a gain medium is pumped by a laser diode (LD, 980 nm) (Connet, Shanghai, China) through a WDM (980/1550 nm) (Connet, Shanghai, China). A polarization-insensitive isolator (PI-ISO) provides unidirectional operation. A polarization controller (PC) (Connet, Shanghai, China) is used to adjust the polarization and balance the gain distribution of the EDF by controlling the cavity loss. The pulses are extracted from the cavity with a 10% output coupler (OC). The PbS QDs SA device is assembled by sandwiching a fiber connector between two FC/PC fiber ferrules. The performance of the laser is recorded by an optical spectrum analyzer (Yokogawa AQ6370D) (Yokogawa, Tokyo, Japan), a second harmonic generation intensity autocorrelator (APE PulseCheck SM1600) (APE, Munich, Germany), an oscilloscope (RIGOL DS4050) (Tektronix, Johnston, OH, USA), and a radio-frequency analyzer (RS-FSV30) (Tektronix, Johnston, OH, USA).

**Figure 4:**
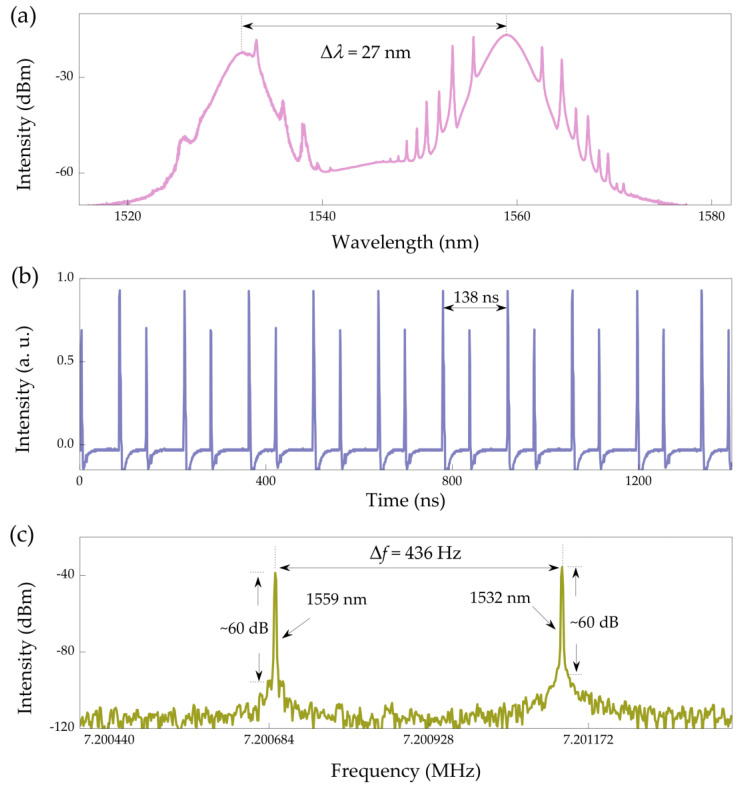
Dual-wavelength conventional solitons. (**a**) Optical spectrum at 1532 and 1559 nm, (**b**) pulse train, and (**c**) corresponding fundamental radio-frequency spectrum.

Based on the above experimental setup, the stable dual-wavelength mode-locked laser pulses are generated when the pump strength is scaled to 200 mW and the PC is finely tuned, as illustrated in Figure 4. The spectrum in Figure 4a appears to be obvious Kelly sidebands, which is a typical feature of the conventional solitons in the anomalous dispersion region [28]. The central wavelengths are 1532 and 1559 nm, and the corresponding 3 dB bandwidths are measured to be 2.8 and 2.5 nm, respectively. The spectrum of dual-wavelength solitons exhibits almost the same peak intensity, and the center wavelength spacing Δ*λ* is 27 nm. Figure 4b illustrates the oscilloscope trace, in which two pulse trains are formed simultaneously. There are two conventional solitons propagating in the laser cavity, and the pulse energy of each soliton varies slightly with the height. Under proper triggering, one pulse sequence stops, and the other moves on the oscilloscope screen. The results show that the two pulse sequences have different group velocities [24]. The corresponding radio-frequency spectrum is demonstrated in Figure 4c. Different from the single-wavelength soliton mode-locked, there are two fundamental frequencies that appear in the dual-wavelength mode-locked spectrum, corresponding to two mode-locked states. The fundamental frequencies are ~7.200695 MHz and ~7.201131 MHz, which are consistent with the mode-locked wavelengths of 1559 and 1532 nm, respectively. The formation of dual-wavelength conventional solitons may be due to the interaction of EDF gain spectrum and cavity-filtering effect [29]. Both of the signal-to-noise ratios are as high as 60 dB, which indicates good temporal stability of the PbS QDs-SA based dual-wavelength mode-locking operation. The frequency interval Δ*f* is 436 Hz. Furthermore, the relationship between Δ*f* and Δ*λ* is theoretically expressed as [30]:

$$\Delta f=\frac{c^{2}D\Delta \lambda }{n^{2}(L+LD\Delta \lambda c/n)}$$

where *L* is the fiber length, *n* is the refractive index of fiber, *D* is the dispersion parameter, and *c* is the speed of light. Here, *L* = 28.5 m, *n* = 1.46, *D_SMF+EDF_* = 10.86 ps/nm/km, and *c* = 3 × 10^8^ m/s. Therefore, the calculated Δ*f* = 434 Hz, which is basically consistent with the experimental results.

**Figure 5:**
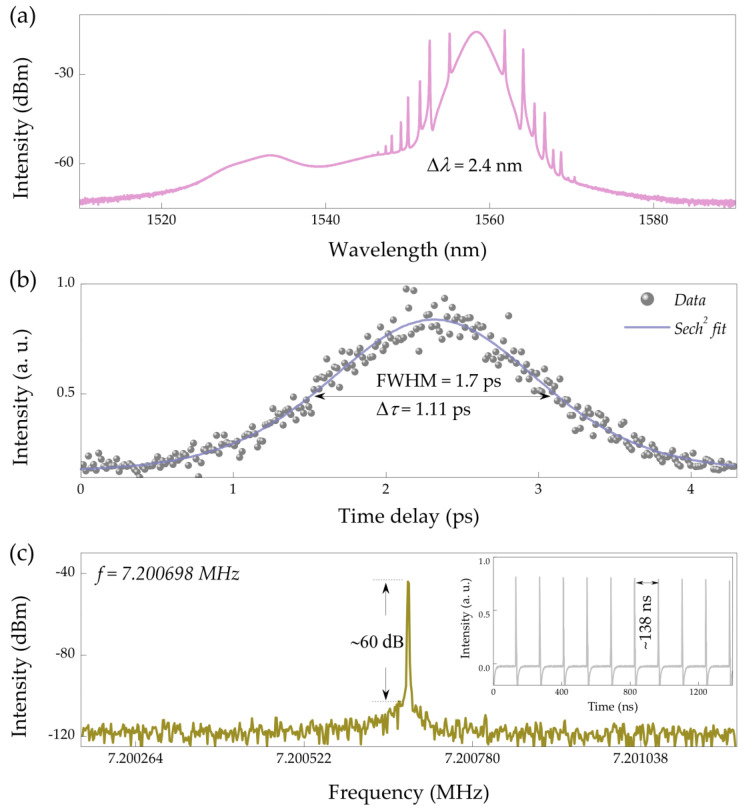
Single-wavelength conventional soliton. (**a**) Optical spectrum at 1559 nm, (**b**) autocorrelation trace, and (**c**) corresponding fundamental radio-frequency spectrum. Inset: pulse train.

By decreasing the pump strength to 80 mW and carefully tuning the PC, dual-wavelength mode locking can be switched into single-wavelength mode locking. The characteristics of the proposed single-wavelength operation are presented in Figure 5. As shown in Figure 5a, the dual-wavelength conventional solitons at 1532 nm disappears, and the single-wavelength soliton remains at 1559 nm. The spectrum exhibits symmetric sid bands, and the 3 dB bandwidth is 2.4 nm. The autocorrelation trace is shown in Figure 5b. The pulse envelope is fitted with a Sech^2^ function. The pulse duration is ~1.11 ps, so that the time-bandwidth product equals to 0.33. Therefore, the single-wavelength conventional soliton is nearly chirp-free. Figure 5c shows the fundamental frequency of ~7.200698 MHz, which is corresponding to a pulse interval of ~138 ns. The signal-to-noise ratio of the soliton is ~60 dB, indicating a stable single-wavelength mode locking. The average output power of the single pulse is 1.9 mW, corresponding to a pulse energy and peak power of ~0.26 nJ and 234 W, respectively.

**Figure 6:**
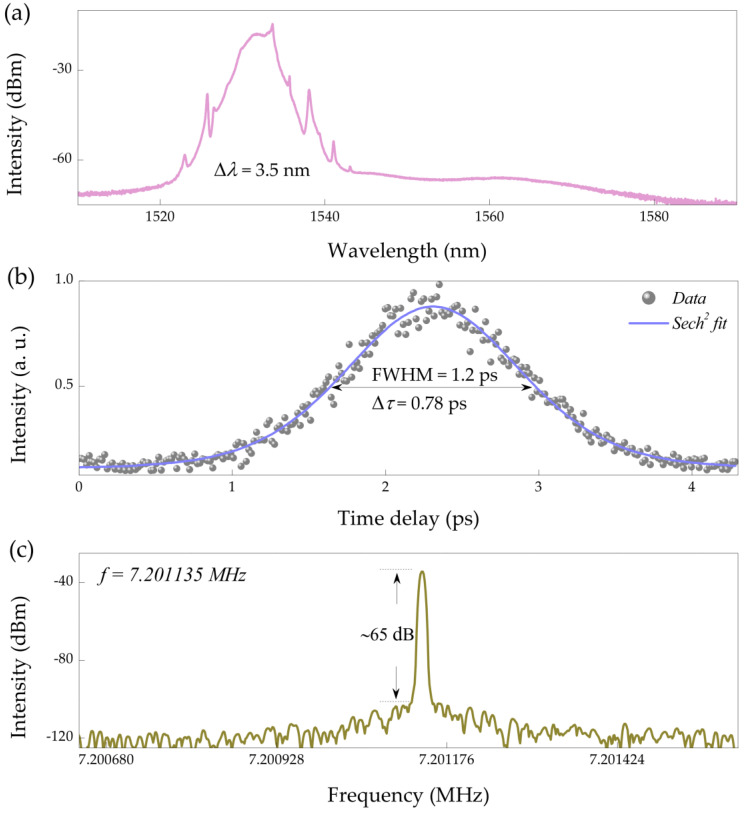
Single-wavelength conventional soliton. (**a**) Optical spectrum at 1532 nm, (**b**) autocorrelation trace, and (**c**) corresponding fundamental radio-frequency spectrum.

When the pump strength increases from 80 to 120 mW, the stable single wavelength mode locking centered at 1532 nm with a 3 dB bandwidth of 3.5 nm is realized, as demonstrated in Figure 6. By Sech^2^ fitting, the pulse duration of the conventional soliton is about 0.78 ps, the corresponding time bandwidth product is calculated to be 0.36, with slight chirp. The fundamental frequency is ~7.201135 MHz, which corresponds to the round-trip time of the cavity length of the fiber laser. The radio-frequency spectrum gives a signal-to-noise ratio of ~65 dB, indicating low-amplitude fluctuations and stable single-wavelength mode-locking state. When the pump power is 500 mW (maximum pump power available of LD in the experiment), the mode-locking operation of the fiber laser is still stable, which shows that the fiber-based PbS QDs SA fiber has good thermal damage. The average output power of the laser cavity is 12 mW, and the corresponding single pulse energy is 1.7 nJ. Therefore, the thermal damage threshold of PbS QDs SA is greater than 30 mJ/cm^2^.

## 4. Conclusions

A passively mode-locked dual-wavelength Er-doped fiber laser is demonstrated with a fiber-based PbS QDs SA. Compared with other nanomaterials, PbS QDs prepared by a modified hot-injection method have the advantages of fast relaxation time, wide band-width, large modulation depth, and thermal damage. Based on this PbS QDs SA, the pulse laser can operate in a dual-wavelength conventional solitons region centered at 1532 and 1559 nm with 3 dB bandwidths of 2.8 and 2.5 nm, respectively. The experimental results reveal that our PbS QDs can be adopted as a broadband SA for application in pulse lasers.

## Data Availability

Not applicable.
